# Weight status misperceptions among UK adults: the use of self-reported vs. measured BMI

**DOI:** 10.1186/s40608-016-0102-8

**Published:** 2016-04-26

**Authors:** Eric Robinson, Melissa Oldham

**Affiliations:** Department of Psychological Sciences, University of Liverpool, Liverpool, L69 7ZA UK

**Keywords:** Weight misperceptions, Body weight norms, Perceived weight, Self-reported BMI

## Abstract

**Background:**

It has been suggested that a significant proportion of overweight and obese individuals underestimate their weight status and think of themselves as being a healthier weight status than they are. The present study examines the prevalence of weight status misperceptions in a recent sample of UK adults, and tests whether the use of self-reported BMI biases estimation of weight status misperceptions.

**Methods:**

Data came from UK adults who took part in the 2013 Health Survey for England. We examined the proportion of overweight vs. normal weight (categorised using self-reported vs. measured BMI) males and females who perceived their weight as being ‘about right’, as well as how common this perception was among individuals whose waist circumference (WC) placed them at increased risk of ill health.

**Results:**

A large proportion of overweight (according to measured BMI) women (31 %) and men (55 %) perceived their weight as being ‘about right’ and over half of participants with a WC that placed them at increased risk of future ill health believed their weight was ‘about right’. The use of self-reported (vs. measured) BMI resulted in underestimation of the proportion of overweight individuals who identified their weight as ‘about right’ and overestimation of the number of normal weight individuals believing their weight was ‘too heavy’.

**Conclusions:**

A large proportion of UK adults who are overweight misperceive their weight status. The use of self-reported BMI data is likely to produce biased estimates of weight status misperceptions. The use of objectively measured BMI is preferable as it will provide more accurate estimates of weight misperception.

## Background

A number of studies have examined whether personal perceptions of weight status correspond with objective weight status. A significant proportion of overweight males and females are thought to underestimate their weight status and believe that their weight ‘is about right’ [1, 2]. Likewise, some studies suggest that a concerning number of healthy weight females believe that their body weight is too heavy [1, 3]. However, estimates of how common under and over estimation of weight status is can vary substantially across studies [4–7].

One explanation of weight status misperceptions is based on body weight norms. For example, Burke, Heiland & Nadler [2] found that fewer US adults identified themselves as being overweight in 2004 than 1994 despite large increases in national rates of obesity during this time. This could be indicative of a generational shift in terms of what is considered to be a normal weight [2]. Moreover, it has been suggested that perception of weight status is more likely to be determined by using those around us as a reference rather than by using clinical recommendations [8] and in line with this overweight and obese teenagers are less likely to think of themselves as being overweight if they have overweight classmates and parents [9]. Furthermore, in recent studies visual exposure to obese males led participants to rate an overweight man as being a healthier [10] and a more normal weight [11] than those exposed to healthy weight males. Thus, it may be that frequent exposure to heavier bodies has led to an upwards shift in terms of what people think a normal weight looks like [12]. This may cause heavier body weights to appear more normal and result in weight status misperceptions [13].

An important unexplored factor that may influence estimates of weight misperception is the use of self-reported vs. measured BMI to categorise participant weight status. Some studies have used objectively measured BMI to draw inferences about weight misperceptions [14–17]. However, there are practical constraints associated with this method and a large number of studies have used self-reported weight to determine the prevalence of weight status misperceptions [6, 7, 13, 18, 19]. This could be problematic as a substantial body of research suggests that BMI is often underestimated when using self-reported data, which can result in underestimation of overweight/obesity [20–22] and overestimation of the proportion of the population who are of a healthy weight. Thus, a potential consequence of using self-reported BMI to examine weight misperception is that it may result in systematic underestimation of the percentage of overweight individuals who misperceive their weight status as being ‘about right’, when they are in fact overweight. Likewise, because self-reported BMI will erroneously categorise some individuals as being of healthy weight, when their weight actually places them in the ‘overweight’ range, it may also result in overestimation of the prevalence of weight misperception amongst healthy weight individuals. Moreover, some studies have indicated that the measurement error associated with self-reported BMI has declined over the last 20 years [23, 24]. This could result in apparent (but erroneous) increases in the prevalence of weight misperceptions. For example, one study reports an increase in underestimation of weight status in women between 2007 and 2012 [6]. However, this may have been in part caused by a larger measurement error in self-reported BMI masking the true prevalence of weight misperceptions at the earlier time point (i.e. making underestimation of weight status among overweight individuals look less common than it actually was).

Although there are general concerns about the accuracy of using self-reported BMI (as opposed to objectively measured BMI) in obesity research, to date no study has examined whether the use of self-reported BMI biases prevalence estimates of weight status misperceptions. Thus, the aim of the present study was to test this hypothesis. In the present study we made use of recently collected data from a large UK study (2013 Health Survey for England; HSE) in order to estimate the prevalence of weight misperceptions among UK adults. As HSE includes measures of self-reported and researcher measured BMI, this allowed us to also examine the hypothesis that the use of self-reported BMI may result in biased estimates of weight misperceptions. We predicted that the use of self-reported BMI may result in an underestimation of the number of overweight individuals who misperceive their weight. Because HSE also includes measured waist circumference, which may be a better indicator of disease risk than BMI [25], we also examined weight perceptions as a function of waist circumference. This allowed us to more thoroughly estimate the prevalence of weight status misperceptions in individuals whose body composition (in terms of waist circumference) places them at an elevated disease risk.

## Method

### Participants

In 2013 a total of 6225 participants (45.2 % male, 54.8 % female) provided self-reported BMI data, objectively measured BMI data and completed the weight perception measure. The sample’s mean age = 49.4 years (SD = 17.7). The majority of participants were Caucasian (90 %) and were employed at the time of the survey (58 %). See Table 1 for detailed information about the sample. As expected, the sample’s mean BMI was lower with self-reported data (mean BMI = 26.3, SD = 5.0) than when objectively measured (mean BMI = 27.4, SD = 5.4) and resulted in fewer classifications of overweight and obesity. See Table 2.

**Table 1:** Sample characteristics

	*N* = 6225
Variable	M (SD)/%
Female (%)	53.8
Age (years)	49.4 (17.7)
Employment (%)^a^	58.0
White (%)	90.0
Education level^b^	2.1 (0.67)
Income (£)^c^	35,139 (29,828)
Health conditions^d^	41.0

^a^Employment: percentage of sample currently in work

^b^Highest education level: 1–3, 1 = no qualification, 2 = below degree, 3 = degree level or equivalent

^c^Income is equivalised according to household size, data shown from 5172 available cases

^d^Health conditions: percentage reporting any physical or mental health conditions/illnesses lasting or expected to last 12 months or more

**Table 2:** Weight status perceptions when using self-reported vs. measured BMI to classify participant weight status

	Self-reported BMI weight status categories				Objective BMI weight status categories			
Weight perception	<18.5	18.5–24.9	25–29.9	30 and above	<18.5	18.5–24.9	25–29.9	30 and above
Females (*n* = 3349)								
Too light	44 (51.2 %)^a^	59 (3.6 %)	1 (0.1 %)	0 (0.0 %)	44 (69.8 %)	59 (4.4 %)	1 (0.1 %)	0 (0.0 %)
About Right	42 (48.8 %)^a^	1245 (75.5 %)^a^	189 (19.3 %)^a^	18 (2.8 %)	19 (30.2 %)	1099 (81.6 %)	342 (30.9 %)	34 (4.1 %)
Too heavy	0 (0.0 %)	345 (20.9 %)^a^	789 (80.6 %)^a^	617 (97.2 %)	0 (0.0 %)	188 (14.0 %)	764 (69.0 %)	799 (95.9 %)
Total	86	1649	979	635	63	1346	1107	833
Males (*n* = 2876)								
Too light	40 (80.0 %)	101 (9.6 %)^a^	9 (0.8 %)	0 (0.0 %)	30 (88.2 %)	107 (13.5 %)	13 (1.0 %)	0 (0.0 %)
About Right	9 (18.0 %)	865 (82.0 %)	505 (42.7 %)^a^	42 (7.1 %)	4 (11.8 %)	653 (82.1 %)	684 (54.7 %)	80 (10.0 %)
Too heavy	1 (2.0 %)	89 (8.4 %)^a^	669 (56.6 %)^a^	546 (92.9 %)	0 (0.0 %)	35 (4.4 %)	553 (44.2 %)	717 (90.0 %)
Total	50	1055	1183	588	34	795	1250	797

^a^Denotes significant difference (*p* < .05) when using self-reported vs. measured BMI to estimate prevalence of weight perception

### Measures

#### Health Survey for England (HSE)

HSE is a yearly household level survey conducted with a nationally representative population sample of English adults. Researcher measured and self-reported weight and height, along with detailed health questionnaires are collected from adults aged 16 or over living at private residential addresses. Participation in the HSE is voluntary. Adults, who are unable to give consent due to mental illness, disability or language barriers, are not included in the survey. Ethical approval for the 2013 study was obtained from the Oxford Research Ethics committee. For detailed information about HSE see [26, 27].

#### Weight, height and waist circumference

During a house visit interview participants were asked to self-report their height and weight. Later in that visit height and weight was measured by a trained researcher. Body mass index (BMI) was calculated (kg/m^2^) and categorised according to World Health Organisation (WHO) guidelines; underweight (BMI < 18.5 kg/m^2^), normal weight (18.5–24.9 kg/m^2^), overweight (25–29.9 kg/m^2^) and obese (30 kg/m^2^ and above). Waist circumference (WC) was also measured by a researcher. Waist circumference was categorised according to WHO guidelines, resulting in three categories; low risk (<94 cm for men or <80 cm for women), increased risk (94–102 cm for men or 80–88 cm for women) and high risk (>102 cm for men or >88 cm for women).

#### Weight perception

During the house visit participants completed a questionnaire item in which they were asked *‘Given your age and height, would you say that you are*’, response options: *about the right weight, too heavy, too light.*

### Analysis

In line with previous studies, normal weight participants were classified as overestimating their weight status if their weight perception response was ‘too heavy’. Overweight and obese participants were classified as having underestimated their weight status if they believed they were ‘about the right weight’. We used a series of chi squares to examine the proportion of participants (underweight, normal weight, overweight and obese participants separately) misperceiving their weight status when self-reported vs. measured BMI was used to characterise weight status. Because of known gender differences in weight status perceptions [2, 4], we conducted analyses in males and females separately.

## Results

### Weight misperceptions: use of measured vs. self-reported BMI

#### Males

Self-reported vs. measured BMI classification of weight status had a significant effect on prevalence of weight misperception among overweight [*x*^2^ = 36.1, *p* < .001] and normal weight [x^2^ = 17.1, *p* < .001], but not underweight [*x*^2^ = 1.3, *p* = .51] or obese males [*x*^2^ = 3.5, *p* = .06]. The use of self-reported BMI, as opposed to measured BMI, resulted in a significant underestimation of the proportion of overweight males who believed their weight was ‘about right’ (42.7 % vs. 54.7 %) and overestimation of the proportion believing their weight was too heavy (56.6 % vs. 44.2 %). Likewise, self-reported BMI resulted in the number of normal weight males believing their weight was too light being underestimated (9.6 % vs. 13.5 %) and the number believing they were too heavy being overestimated (8.4 % vs. 4.4 %). See Table 2.

#### Females

Self-reported vs. measured BMI classification of weight status had a significant effect on prevalence of weight misperception among overweight [*x*^2^ = 36.7, *p* < .001], normal weight [*x*^2^ = 24.9, *p* < .001] and underweight [*x*^2^ = 5.2, *p* = .02], but not obese females [*x*^2^ = 1.6, *p* = .20]. The use of self-reported BMI, as opposed to measured BMI, resulted in a significant underestimation of the proportion of overweight females who believed their weight was ‘about right’ (19.3 % vs. 30.9 %) and overestimation of the proportion believing their weight was too heavy (80.6 % vs. 69.0 %). Self-reported BMI was also associated with an overestimation in the proportion of normal weight females who believed they were too heavy (20.9 % vs. 14.0 %) and underestimated the proportion believing they were ‘about right’ (75.5 % vs. 81.6 %). Among underweight females, self-reported BMI also resulted in an overestimation in the proportion of females who believed their weight was about right (48.8 % vs. 30.2 %). See Table 2.

### Additional data

#### Waist circumference

Approximately 50 % of males and females with weight circumferences placing them at increased risk of ill health believed their weight was ‘about right’. Around 19 % of participants whose waist circumference was in the high risk category perceived their weight as being ‘about right’. See Table 3. We also examined the proportion of participants who had an ‘overweight’ or ‘obese’ BMI and waist circumference which placed them at increased or high risk of ill health. See online supplemental materials. The percentages of participants believing their weight was ‘about right’ were still relatively high; for example, among overweight participants whose waist circumference was classed as ‘high risk’, 31 % underestimated their weight status as being ‘about right’. *Age:* For perceptions of weight status according to BMI grouping and age, see online supplemental materials.

**Table 3:** Weight status perceptions according to waist circumference group

	Females			Males		
Weight perception	Low risk (<80 cm)	Increased risk (80–88 cm)	High risk (>88 cm)	Low risk (<94 cm)	Increased risk (94–102 cm)	High risk (>102 cm)
Too light	69 (8.0 %)	8 (1.2 %)	1 (0.1 %)	95 (10.9 %)	12 (2.1 %)	0 (0.0 %)
About Right	665 (76.8 %)	348 (51.0 %)	244 (17.6 %)	675 (77.6 %)	310 (53.4 %)	180 (19.6 %)
Too heavy	132 (15.2 %)	327 (47.9 %)	1140 (82.3 %)	100 (11.5 %)	259 (44.6 %)	740 (80.4 %)
Total	866	683	1385	870	581	920

*n* = 5305 participants

## Discussion

In the present study we made use of recently collected data to examine the prevalence of weight status misperceptions in a sample of UK adults. Using measured BMI, we found that a large proportion of overweight males (55 %) and females (31 %) perceived their weight as being ‘about right’, although underestimation of weight status was less common among obese participants (≤10 %). Moreover, using waist circumference as a measure of risk of future ill health, around half of males and females at increased risk and almost one fifth of those at high risk, perceived their weight status as being ‘about right’. Even when ill health risk profiles were combined (e.g. participants with overweight or obese BMI and raised weight circumference) a substantial percentage of individuals underestimated their weight as being ‘about right’. These findings may have public health implications, as weight status misperceptions are thought to be associated with a variety of health relevant outcomes, such as weight loss intentions, physical activity and mental well-being [3, 5, 18, 19]. The present findings are consistent with the notion that heavier body weights may have now become ‘normalised’ as a result of increased obesity prevalence. It has been previously suggested that this process of normalisation may have increased the prevalence of weight status misperceptions among overweight and obese individuals [2, 11]. Moreover, it is plausible that larger body sizes may have now become more acceptable due to the increased prevalence of obesity [12].

In the present study we also examined whether the use of self-reported BMI, as opposed to objectively measured BMI, produces measurement error when estimating the prevalence of weight status misperceptions. The use of self-reported BMI resulted in an underestimation of the number of overweight individuals misperceiving their weight (‘about right’) and overestimated the number of healthy weight individuals who misperceived their weight status as being ‘too heavy’. In some instances this measurement error was sizeable. For example, the use of self-reported BMI resulted in approximately only 19 % of overweight females underestimating their weight status, when in reality 31 % did so. This is of importance as a large number of studies have used only self-reported BMI data to estimate both the prevalence of weight status misperceptions and their possible consequences [6, 7, 13, 18, 19]. Moreover, because the degree of underestimation of BMI caused by self-reported weight and height may change over time [23, 24], it will be important for studies which attempt to track longitudinal changes in weight misperceptions to adjust for this potential confound or rely on objective measures of adiposity, which has not always been the case to date [6, 7]. Our findings also support recent suggestions that objective measures of adiposity are required when examining weight status misperception [28]. There are of course practical constraints associated with collecting objective measures of adiposity and this may have contributed to the reliance on self-report measures of BMI in studies examining weight perceptions [6, 7, 13, 18, 19]. Nonetheless, where possible we strongly suggest that future research should make use of objectively measured BMI. If this is not at all feasible, then it will be important to consider the likelihood that the estimates of weight misperceptions produced using self-reported BMI will be biased and underestimate the frequency by which overweight individuals misperceive their weight status.

### Limitations

Because a small proportion of participants provided self-reported BMI, but not measured BMI, we were unable to include them in analyses and these participants’ measured BMI may have differed to the overall sample. Participants reported their weight status as being ‘too light’ ‘about right’ or ‘too heavy’ and it is feasible that if a wider range of response options had been available (i.e. slightly overweight), the tendency for overweight participants to underestimate their weight status may have been reduced. Participants also reported their personal perceptions of weight status without the use of a visual aid. A number of studies have made use of line drawings or body silhouettes to understand participants’ visual perceptions of personal weight status (e.g. [29]) and it would have been informative to have included both types of measure in the present study. A further limitation of the present study was that our sample was predominantly of white/Caucasian ethnicity (90 %), so we are not able to draw conclusions about weight perceptions in other ethnic groups. This is of importance, as a number of studies suggest that ethnicity may be an important factor which predicts whether a person misperceives their weight. For example underestimation of personal overweight and obesity is more common among black individuals than white participants [30, 31].

## Conclusions

A large proportion of UK adults who are overweight misperceive their weight status. The use of self-reported BMI data is likely to produce biased estimates of weight status misperceptions. The use of objectively measured BMI is preferable as it will provide more accurate estimates of weight misperception.

### Ethics approval and consent to participate

Ethical approval for the 2013 study was obtained from the Oxford Research Ethics committee.

### Consent for publication

Not applicable.

### Availability of data

HSE data is accessible online (https://data.gov.uk/dataset/health_survey_for_england). UK University staff and members of the UK Access Management Federation can access and download the data using their institutional log in. Other users can access the data but would need to apply for a UK data archive username and password. Intended use of data must be registered.

## Abbreviations

BMI: body mass index
HSE: Health Survey for England
WC: waist circumference
WHO: World Health Organisation

## References

[1] Brug J, Wammes B, Kremers S, Giskes K, Oenema A (2006). Underestimation and overestimation of personal weight status: associations with socio-demographic characteristics and weight maintenance intentions. J Hum Nutr Diet.

[2] Burke MA, Heiland FW, Nadler CM (2010). From “overweight” to “about right”: Evidence of a generational shift in body weight norms. Obesity.

[3] Sutin AR, Terracciano A (2015). Body weight misperception in adolescence and incident obesity in young adulthood. Psychol Sci.

[4] Andrade FCD, Raffaelli M, Teran-Garcia M, Jerman JA, Garcia CA (2012). Weight status misperception among Mexican young adults. Body Image.

[5] Duncan DT, Wolin KY, Scharoun-Lee M, Ding EL, Warner ET, Bennett GG (2011). Does perception equal reality? Weight misperception in relation to weight-related attitudes and behaviors among overweight and obese US adults. Int J Behav Nutr Phys Act.

[6] Johnson F, Beeken RJ, Croker H, Wardle J (2014). Do weight perceptions among obese adults in Great Britain match clinical definitions? Analysis of cross-sectional surveys from 2007 and 2012. BMJ Open.

[7] Matthiessen J, Biltoft-Jensen A, Fagt S, Knudsen VK, Tetens I, Groth MV (2014). Misperception of body weight among overweight Danish adults: trends from 1995 to 2008. Public Health Nutr.

[8] Ali MM, Amialchuk A, Renna F (2011). Social network and weight misperception among adolescents. South Econ J.

[9] Maximova K, McGrath JJ, Barnett T, O’Loughlin J, Paradis G, Lambert M (2008). Do you see what I see? Weight status misperception and exposure to obesity among children and adolescents. Int J Obes.

[10] Oldham M, Robinson E. Visual weight status misperceptions of men: Why overweight can look like a healthy weight. J Health Psychol. 2015;20:1-10. 10.1177/1359105314566257 25609407

[11] Robinson E, Kirkham TC (2014). Is he a healthy weight? Exposure to obesity changes perception of the weight status of others. Int J Obes.

[12] Robinson E, Christiansen C (2014). The changing face of obesity; Exposure to and acceptance of obesity. Obesity.

[13] Robinson E, Hogenkamp PS. Visual perceptions of male obesity: a cross-cultural study examining male and female lay perceptions of obesity in Caucasian males. BMC Public Health. 2015;15:492-501. 10.1186/s12889-015-1821-3 PMC4438518 25981526

[14] Lemon SC, Rosal MC, Zapka J, Borg A, Anderson V (2009). Contributions of weight perceptions to weight loss attempts: Differences by body mass index and gender. Body Image.

[15] Malinauskas BM, Raedeke TD, Aeby VG, Smith JL, Dallas MB (2006). Dieting practices, weight perceptions and body composition: A comparison of normal weight, overweight and obese college females. Nutr J.

[16] Gillison FB, Standage M, Skevington SM, Relationships among adolescents weight perceptions, exercise goals, exercise motivation, quality of life and leisure-time exercise behaviour: a self determination theory approach. *Health Education Research*. 2006; 21(6):836–847. 10.1093/her/cyl139 17101718

[17] Zimmerman MB, Hess, SY, Hurrell RF. A national study of the prevalence of overweight and obesity in 6–12 year old Swiss children: body mass index, body-weight perceptions and goals. *European Journal of Clinical Nutrition*. 2000;54(7):568–572 10.1038/sj.ejcn.1601058 10918467

[18] Atlantis E, Ball K (2008). Association between weight perception and psychological distress. Int J Obes.

[19] Atlantis E, Barnes EH, Ball K (2008). Weight status and perception barriers to healthy physical activity and diet behavior. Int J Obes.

[20] Bae J, Joung H, Kim JY, Kwon KN, Kim Y, Park SW (2010). Validity of self-reported height, weight, and body mass index of the Korea Youth Risk Behavior Web-based Survey questionnaire. J Prev Med Public Health.

[21] Nyholm M, Gullberg B, Merlo J, Lundqvist-Persson C, Rastam L, Lindblad U (2007). The validity of obesity based on self-reported weight and height: Implications for population studies. Obesity.

[22] Rowland ML (1990). Self-reported weight and height. Am J Clin Nutrn.

[23] Stommel M, Osier N (2013). Temporal changes in bias of body mass index scores based on self-reported height and weight. Int J Obes.

[24] Connor Gorber S, Tremblay MS (2010). The bias in self-reported obesity from 1976 to 2005: a Canada-US comparison. Obesity.

[25] Janssen I, Katzmarzyk PT, Ross R (2004). Waist circumference and not body mass index explains obesity-related health risk. Am J Clin Nutr.

[26] Mindell J, Biddulph JP, Hirani V, Stamatakis E, Craig R, Nunn S, Shelton N (2012). Cohort profile: the health survey for England. Int J Epidemiol.

[27] Oyebode O, Mindell J (2013). Use of data from the health survey for England in obesity policy making and monitoring. ObesRevs.

[28] Lewis DW, Dutton GR, Affuso O (2015). Physical characteristics associated with weight misperception among overweight and obese men: NHANES 1999–2006. Obesity.

[29] Lynch EB, Kane J (2014). Body size perception among African American women. J Nutr Educ Behav.

[30] Dorsey RR, Eberhardt MS, Ogden CL (2009). Racial/ethnic differences in weight perception. Obes.

[31] Hendley Y, Zhao L, Coverson DL, Din-Dzietham R, Morris A, Quyumi AA, Gibbons GH, Vaccarino V (2011). Differences in weight perception among blacks and white. J Women’s Health.

